# Impact of JH Signaling on Reproductive Physiology of the Classical Insect Model, *Rhodnius prolixus*

**DOI:** 10.3390/ijms232213832

**Published:** 2022-11-10

**Authors:** Jimena Leyria, Ian Orchard, Angela B. Lange

**Affiliations:** Department of Biology, University of Toronto Mississauga, Mississauga, ON L5L 1C6, Canada

**Keywords:** Juvenile hormone signaling, reproduction, *Rhodnius prolixus*, Methoprene tolerant, Taiman, Krüppel homolog 1

## Abstract

In adult females of several insect species, juvenile hormones (JHs) act as gonadotrophic hormones, regulating egg production. JH binds to its nuclear receptor, Methoprene tolerant (Met), triggering its dimerization with the protein Taiman (Tai). The resulting active complex induces transcription of JH response genes, such as Krüppel homolog 1 (Kr-h1). In this study we report for the first time the participation of the isoform JH III skipped bisepoxide (JHSB_3_) and its signaling pathway in the reproductive fitness of the classical insect model *Rhodnius prolixus*. The topical application of synthetic JHSB_3_ increases transcript and protein expression of yolk protein precursors (YPPs), mainly by the fat body but also by the ovaries, the second source of YPPs. These results are also confirmed by ex vivo assays. In contrast, when the JH signaling cascade is impaired via RNA interference by downregulating *RhoprMet* and *RhoprTai* mRNA, egg production is inhibited. Although *RhoprKr-h1* transcript expression is highly dependent on JHSB_3_ signaling, it is not involved in egg production but rather in successful hatching. This research contributes missing pieces of JH action in the insect model in which JH was first postulated almost 100 years ago.

## 1. Introduction

Juvenile hormones (JHs) are one of the most famous hormonal factors responsible for the control of critical developmental events in insects. Primarily produced and secreted from an endocrine gland named the corpus allatum (CA), JH was originally postulated by Wigglesworth [1] and shown to regulate metamorphosis in the kissing bug *Rhodnius prolixus*, and subsequently reported to control ovarian maturation in adult females and accessory gland development in adult males [2]. This pioneering research on *R. prolixus* reproductive physiology was further explored by Davey and colleagues [3,4] using classical endocrinological techniques and JH analogs. It is stated that the foundations of insect endocrinology were born using this historically important model insect, in which a JH was first postulated almost 100 years ago. Despite this early research, the mechanisms and target genes underlying JH action in *R. prolixus* are not understood. Indeed, although seven epoxidated JH homologs and a non-epoxidated methyl farneosate (MF) have been described in different arthropods in the last 50 years [5], the true JH structure in *R. prolixus* was only recently discovered; screening for several JH homologs in the hemolymph of *R. prolixus* revealed only the presence of the isoform JH III skipped bisepoxide (JHSB_3_) [6].

Interestingly, a JH receptor involved in patency, i.e., shrinkage of follicle cells allowing access of the oocyte to hemolymph vitellogenin (Vg), was also first postulated using *R. prolixus* females as a model. The identity of the receptor is still unknown, but may be a membrane receptor responsible for the activation of a JH-sensitive sodium–potassium ATPase via a protein kinase C signaling pathway [3]. There is, however, evidence that JH directly modulates gene expression in other insects. The discovery of the gene Methoprene-tolerant (Met) as a putative JH nuclear receptor in *Drosophila melanogaster* was a milestone event for understanding JH signaling transduction [7], although this hypothesis was only confirmed 20 years after its report, using another insect model, the red flour beetle *Tribolium castaneum* [8]. Met is a member of the basic-helix-loop-helix (bHLH)-Period-Aryl hydrocarbon receptor nuclear translocator-Single-minded (PAS) domain family. Typically, these factors form heterodimers with other members of this protein family to activate transcription. Before the interaction with JH, Met is present as a homodimer complex, but upon hormone binding, Met heterodimerizes with Taiman (Tai). Tai is a steroid receptor coactivator counterpart of the mammalian steroid receptor coactivator 1, also known as “FISC” in the yellow fever mosquito *Aedes aegypti* or “Src” in *T. castaneum* [9,10], and forms an active complex that modulates the transcription of several target genes by binding to their JH response elements (JHRE). The zinc-finger transcription factor Krüppel homolog 1 (Kr-h1) is a well described gene transcriptionally activated by the JH-Met-Tai complex. In *T. castaneum*, Met-Tai complex binds E-boxes in the upstream region of Kr-h1 gene to promote its transcription [11,12]. Kr-h1 exerts a dual regulatory role; on the one hand preventing precocious metamorphosis in immature stages, and on the other hand stimulating aspects of adult reproduction in some insect species [13,14,15,16,17,18,19]. In *R. prolixus*, Kr-h1 regulates metamorphosis [20], however, its role in reproductive physiology is less understood. Although the presence of Met has already been reported in *R. prolixus* [21], the downstream signaling pathway resulting in the synthesis of yolk protein precursors (YPPs) by the fat body, a multifunctional organ analogous to vertebrate adipose tissue and liver, remains unknown. Remarkably, *R. prolixus* follicle cells themselves can also synthesize the main YPP, Vg (RhoprVg) [22], although it is unknown if this synthesis is under JH control. 

*Rhodnius prolixus* has not only been the historic model for studying reproduction in insects [23], but also it is medically important for being a vector of the parasite, *Trypanosoma cruzi*, the causative agent of Chagas disease in humans, an infection endemic in Latin American countries. Understanding key events involved in the reproductive biology of *R. prolixus* is of some critical importance in the control of the transmission of the disease, especially considering the concern that endemic areas may expand due to climate change [24]. Here, we elucidate the mechanisms and target genes underlying JH action, identifying the genes *Tai* and *Kr-h1* in *R. prolixus* (*RhoprTai* and *RhoprKr-h1*, respectively), and reporting the physiological roles of these components in the JH signaling cascade. *RhoprMet* and *RhoprTai* transcript levels are not influenced by blood feeding, but the transcript levels for *RhoprKr-h1*, *RhoprVg* and other YPPs are. Topical application of synthetic JHSB_3_ to females increases transcript and protein expression of *RhoprVg*, mainly in the fat body but also in the ovaries, the second source of YPPs. These results are confirmed by ex vivo assays and by downregulating *RhoprMet* and *RhoprTai* transcripts using RNA interference (RNAi). When the JHBS_3_ signaling cascade is impaired, the synthesis of YPPs decreases and ovarian follicles do not take up nutrients, resulting in an inhibition of egg production. The results reveal that follicular synthesis of YPPs is also dependent on JHSB_3_ signaling and expose a major participation of not only RhoprMet but also RhoprTai in successful reproduction. When the JH response transcript *RhoprKr-h1* is downregulated, vitellogenesis is not affected, but aspects of successful hatching are. 

This study fills important knowledge gaps of JH action in the important historical insect model used by Wigglesworth and Davey, *R. prolixus*. 

## 2. Results

### Subsections

- *RhoprMet* and *RhoprTai* transcripts are constitutively expressed in *R. prolixus* but *RhoprKr-h1* appears to be a nutritional response gene, as are the yolk protein precursors.

**Figure 1:**
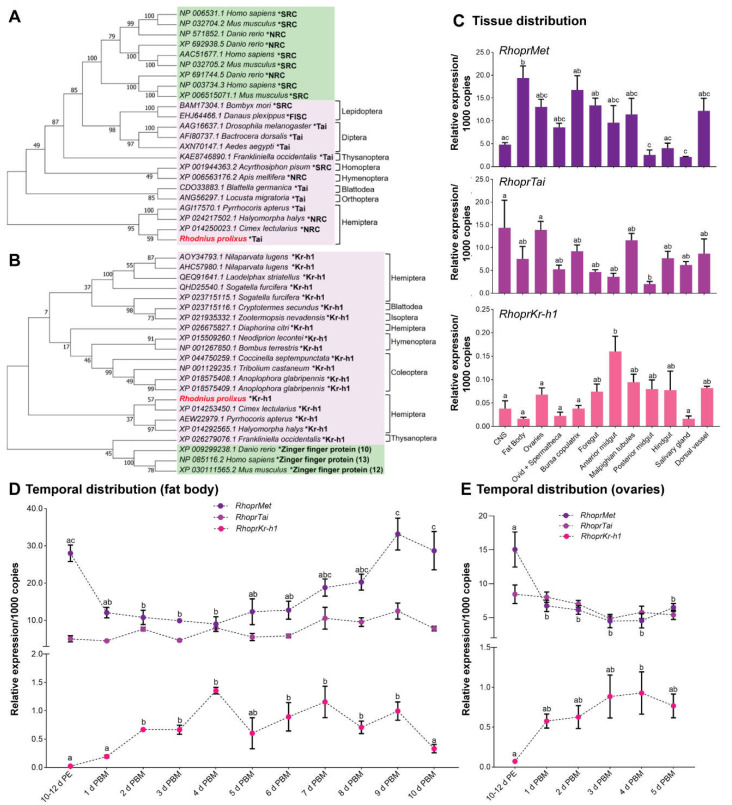
Identification of RhoprTai and RhoprKr-h1 and its transcript expression. (**A**,**B**) Phylogenetic relationship of RhoprTai (**A**) and RhoprKr-h1 (**B**) from insects (lilac boxes) and mammals (green boxes). The evolutionary history was inferred by using the Maximum Likelihood method. The percentage of replicate trees in which the associated taxa clustered together in the bootstrap test (1000 replicates) are shown next to the branches. Protein sequences (*) are labelled by species and order name, and identified with their GenBank accession number. (**C**) Distribution of *RhoprMet*, *RhoprTai* and *RhoprKr-h1* transcripts in unfed adult female *R. prolixus*; (**D**,**E**) Temporal transcript expression of *RhoprMet*, *RhoprTai* and *RhoprKr-h1* in the fat body (**D**) and ovaries (**E**) at 10–12 d post ecdysis (d PE) and throughout several days post blood meal (d PBM). The transcript levels were quantified using RT-qPCR and analyzed by the 2^−ΔCt^ method. The y axes represent the relative expression obtained via geometric averaging using *Rp49*, *18S rRNA* and *actin* as reference genes. The results are shown as the mean ± SEM (*n* = 4–5, where each *n* represents a pool of tissues from 3 insects). Different letters indicate significant differences at *p* < 0.05 (One-way ANOVA and Tukey’s test as the post hoc test). *RhoprTai* had no significant difference between all times tested (*p* < 0.05). SRC, steroid receptor coactivator family proteins; NRC, nuclear receptor coactivator; FISC, βFtz-F1 Interacting Steroid receptor Coactivator; CNS, central nervous system; Ovid, oviducts.

Based on the *R. prolixus* genome database and using BLAST tools, we found high-confident hits for RhoprTai and RhoprKr-h1 protein sequences. Our phylogenetic analysis reveals that these sequences are closely related to those from the hemipteran *Cimex lectularius*, and both are grouped with other insects of the same order (Figure 1A,B, lilac boxes). The related sequences from mammals form a separate monophyletic cluster, with a distribution that was consistent with the taxonomy established for the corresponding orders (Figure 1A,B, green boxes). The existence of two RhoprMet isoforms produced by splicing mechanisms can be predicted from the *R. prolixus* genome [21,25]. In spite of the efforts made, the *RhoprMet2* mRNA expression could not be analyzed individually in this work. Our analysis to reveal the tissue distribution and temporal mRNA expression was done using a small sequence shared between both RhoprMet1 and RhoprMet2, and therefore represents total RhoprMet. However, since total *RhopMet* (RhoprMet1 + RhoprMet2) is not statistically different from *RhoprMet1*, total *RhoprMet* is predominantly represented by *RhoprMet1* (Supplementary Figure S1). Tissue-specific transcript expression of *RhoprMet*, *RhoprTai* and *RhoprKr-h1* was investigated in unfed adult females (baseline condition, where JH titers in the hemolymph are supposed to be low) (Figure 1C). While transcript levels are quantifiable in all tissues analyzed, they are typically observed higher in those tissues involving in ion-transporting epithelia (i.e., Malpighian tubules and anterior midgut), but *RhoprMet* and *RhoprTai* are also higher in tissues related with reproductive physiology, i.e., central nervous system (CNS), ovaries and fat body. After a blood meal (the main stimulus for vitellogenesis and egg growth), *RhoprMet* levels decrease, *RhoprTai* remains constant and *RhoprKr-h1* levels increase, not only in the fat body but also in the ovaries (Figure 1D,E). *RhoprKr-h1* expression shows a similar pattern to those given by the YPPs, *RhoprVg* (RhoprVg1 and RhoprVg2), *Rhodnius heme binding protein* (RHBP) and *lipophorin* (RhoprLp) (Supplementary Figure S2A,B), which show a statistically significant increase as the days after a blood meal progress. However, in ovaries, YPPs mRNA expression is always significantly lower relative to fat body. In ovaries, the transcript expression of endocytic receptors, i.e., *Lp receptor* (RhoprLpR) and *Vg receptor* (RhoprVgR), slightly decreases in insects recently fed (Supplementary Figure S2A,B). Interestingly, *RhoprMet* and *RhoprTai* mRNA levels are more than 20-fold higher with respect to the low, but measurable, *RhoprKr-h1* levels, in all the tissues and time points examined (Figure 1C–E).

- *RhoprKr-h1* mRNA is a JHSB_3_-dependent gene, as are the yolk protein precursors

**Figure 2:**
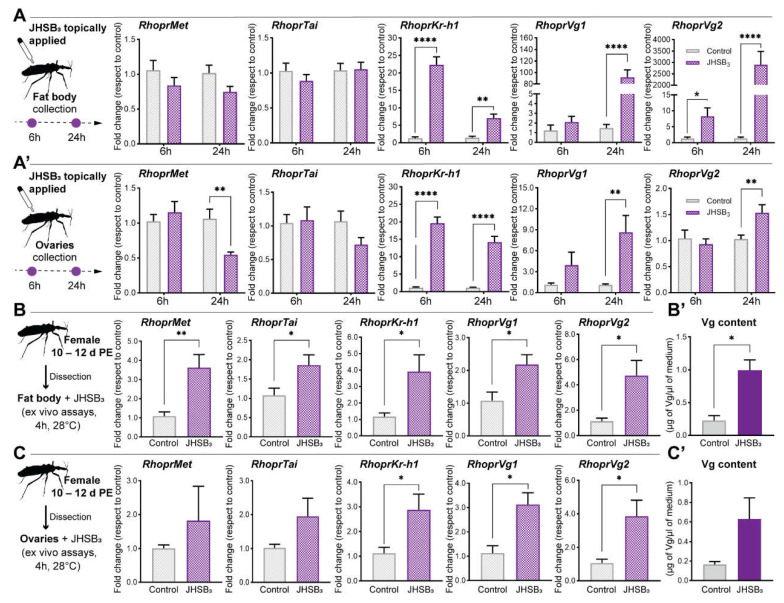
Effect of exogenous JHSB_3_ treatment. (**A**,**A’**) In vivo assays: JHSB_3_ treatment on *RhoprMet, RhoprTai, RhoprKr-h1* and *RhoprVg* mRNA expression in the fat body (**A**) and ovaries (**A’**). JHSB_3_ (50 pg in 10 µL of acetone) was topically applied in newly emerged adult females, and transcript levels were measured 6 and 24 h later. The results are shown as the mean ± SEM (*n* = 5–6, where each *n* represents an individual tissue from 1 insect). Transcript expression was quantified using RT-qPCR and analyzed the 2^−ΔΔCt^ method. The y axes represent fold change in expression relative to control (acetone, value ~1) obtained via geometric averaging using *Rp49* and *actin* as reference genes. ** *p* < 0.01; **** *p* < 0.0001 (Student’s *t*-test). (**B**,**C**) Ex vivo assays: JHSB_3_ treatment on *RhoprMet*, *RhoprTai*, *RhoprKr-h1* and *RhoprVg* mRNA expression in the fat body (**B**) and ovaries (**C**). JHSB_3_ was dissolved in acetone, and then added to the incubation medium (final concentration: 35 nM) containing an individual fat body or ovary, as indicated. The results are shown as the mean ± SEM (*n* = 5–6, where each *n* represents an individual tissue from 1 insect). Transcript expression was quantified using RT-qPCR and analyzed the 2^−ΔΔCt^ method. The y axes represent fold change in expression relative to control (acetone, value ~1) obtained via geometric averaging using *Rp49* and *actin* as reference genes. * *p* < 0.05; ** *p* < 0.01 (Student’s *t*-test). (**B’,C’**) Vitellogenin (Vg) content in the incubation medium was measured by ELISA. The results are shown as the mean ± SEM (*n* = 5–6, where each *n* represents the hemolymph from 1 insect). * *p* < 0.05 (Student’s *t*-test).

To test whether *RhoprMet*, *RhoprTai* and *RhoprKr-h1* are JH-inducible genes, we treated newly emerged adult females with exogenous JHSB_3_, the naturally occurring JH homolog in *R. prolixus*, and measured mRNA levels 6 and 24 h after treatment in the fat body and ovaries (Figure 2A,A’, respectively). *RhoprMet* and *RhoprTai* are not up-regulated by JHSB_3_ in the fat body and ovaries (*p* > 0.05, *n* = 5–6); indeed, *RhoprMet* transcript levels tend to decrease, as was shown after a blood meal, when JHSB_3_ titers are supposed to be increasing. On the other hand, *RhoprKr-h1* is an early JHSB_3_-response gene, as the mRNA levels are up-regulated 6 h after treatment in the fat body and ovaries (*p* < 0.0001, *n* = 5–6). As expected, *RhoprVg1* and *RhoprVg2* mRNA in the fat body (*p* < 0.0001, *n* = 5–6, at 24 h) and ovaries (*p* < 0.01, *n* = 5–6, at 24 h) (Figure 2A,A’), as well as *RHBP* (*p* < 0.01, *n* = 5, at 24 h) and *RhoprLp* (*p* < 0.05, *n* = 5, at 24 h) mRNA in the fat body (Supplementary Figure S3A), are also positively regulated by JHSB_3_ with the stimulatory effect increasing over time, being higher 24 h after treatment. *RhoprLpR* and *RhoprVgR* are down-regulated at this time in the ovaries (*p* < 0.05, *n* = 5) (Supplementary Figure S3B), as was shown after feeding (Supplementary Figure S2C). The exogenous hormone stimulation is also capable of significantly increasing the release of Vg into hemolymph (*p* < 0.05, *n* = 5) (Supplementary Figure S3C).

As an in vivo stimulation is systemic, the possibility of an indirect effect on regulation of JH-dependent genes cannot be completely ruled out; thus, we confirmed the effect of JHSB_3_ on the transcript expression of *RhoprMet*, *RhoprTai*, *RhoprKr-h1*, YPPs and endocytic receptors by ex vivo assays. Interestingly, when the fat body is incubated separately in a medium containing exogenous JHSB_3_, an up-regulation of *RhoprMet* (*p* < 0.01, *n* = 5–6) and *RhoprTai* (*p* < 0.05, *n* = 5–6) mRNA is observed (Figure 2B), which was not seen following feeding (Figure 1D). In these ex vivo experiments, *RhoprKr-h1* and YPP mRNA levels significantly increase with respect to controls in both, the fat body and ovaries (*p* < 0.05, *n* = 5–6) (Figure 2B,C and Supplementary Figure S4A), whereas *RhoprLpR* and *RhoprVgR* are down-regulated in the ovaries (Supplementary Figure S4B), as was shown after feeding (Figure 1E). The levels of Vg content in the incubation medium were also quantified, and whereas only traces of Vg are measured in control media, the fat body and ovaries release significant amounts of Vg into the incubation medium after hormone treatment (Figure 2B’,C’, respectively).

- JHSB_3_ target genes are controlled through Met-Tai complex

**Figure 3:**
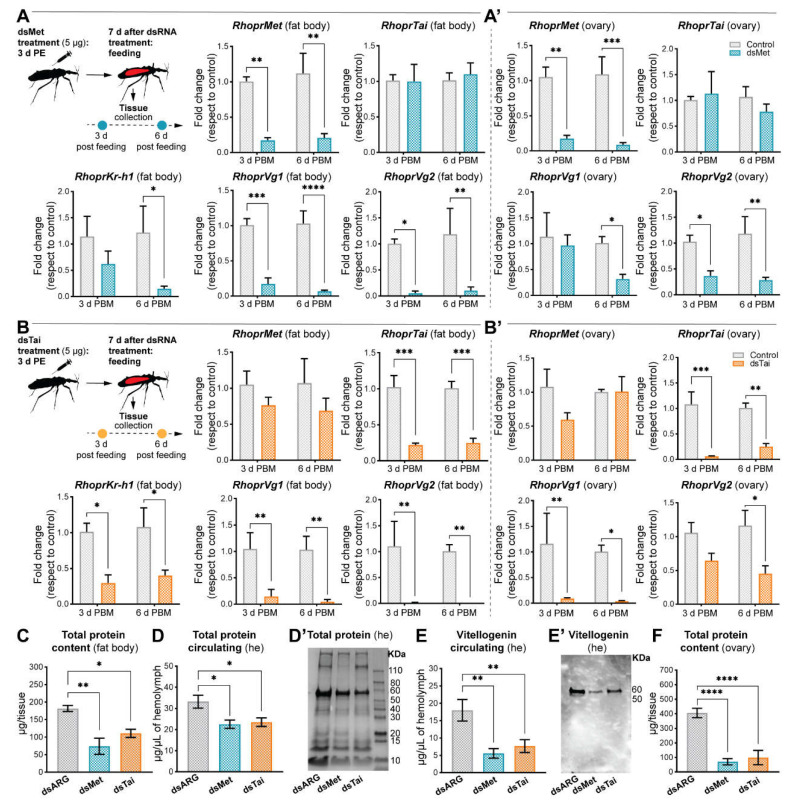
Effect of dsRNA treatment at different time points during vitellogenesis. (**A**,**A’**) *RhoprMet*, *RhoprTai*, *RhoprKr-h1* and *RhoprVg* mRNA expression in the fat body (**A**) and ovaries (**A’**) of dsMet-injected females at 3 days post blood meal (3 d PBM) and 6 d PBM (**B**,**B’**) *RhoprMet*, *RhoprTai*, *RhoprKr-h1* and *RhoprVg* mRNA expression in the fat body (**B**) and ovaries (**B’**) of dsTai-injected females at 3 d PBM and 6 d PBM. Transcript levels were quantified using RT-qPCR and analyzed by the 2^-ΔΔCt^ method. The y axes represent the fold change in expression relative to control (dsARG, value ~ 1) obtained via geometric averaging using *Rp49* and *actin* as reference genes. The results are shown as the mean ± SEM (*n* = 5–6, where each *n* represents an individual tissue from 1 insect). * *p* < 0.05; ** *p* < 0.01; *** *p* < 0.001; **** *p* < 0.0001 (Student’s *t*-test). (**C**) Protein content in the fat body of dsRNA-injected females at 4 d PBM. The results are shown as the mean ± SEM (*n* = 5–6, where each *n* represents the fat body from 1 insect). * *p* < 0.05; ** *p* < 0.01 (One-way ANOVA and Tukey’s test as the post hoc test). (**D**) Total protein circulating in dsRNA-injected females at 4 d PBM. The results are shown as the mean ± SEM (*n* = 5–6, where each *n* represents hemolymph from 1 insect). * *p* < 0.05 (One-way ANOVA and Tukey’s test as the post hoc test). (**D’**) SDS-PAGE analysis of hemolymph (1 μL) after silencing of *RhoprMet* and *RhoprTai*. Image representative of 3 independent experiments. (**E**) Quantification of vitellogenin (Vg) circulating in the hemolymph of dsRNA-injected females by ELISA. The results are shown as the mean ± SEM (*n* = 5–6, where each *n* represents hemolymph from 1 insect). ** *p* < 0.01 (One-way ANOVA and Tukey’s test as the post hoc test). (**E’**) Western blot image showing Vg circulating in the hemolymph of dsRNA-injected females (1 μL of hemolymph in 1:20 dilution). Image representative of 3 independent experiments. (**F**) Total protein content in the ovaries of dsRNA-injected females at 4 d PBM. The results are shown as the mean ± SEM (*n* = 5–6, where each *n* represents ovaries from 1 insect. **** *p* < 0.0001 (One-way ANOVA and Tukey’s test as the post hoc test). he, hemolymph.

In order to analyze not only the effects of RhoprMet and RhoprTai on the known JHSB_3_-target genes, but also the individual contribution of each, we individually silenced both components of the receptor complex. The effects were tested at two time points during vitellogenesis; early vitellogenesis (3 days post feeding) and just prior to oviposition (6 days post feeding). The efficiency of RNAi for each factor is between 70–80% in both tissues (the fat body and the ovaries) and time points assayed. Depletion of *RhoprMet* or *RhoprTai* transcripts significantly decreases *RhoprKr-h1* (*p* < 0.05, *n* = 5–6), *RhoprVg1* (*p* < 0.0001 for dsMet and *p* < 0.01 for dsTai, *n* = 5–6) and *RhoprVg2* (*p* < 0.01, *n* = 5–6) mRNA levels in the fat body at 6 days post blood meal (Figure 3A,B). These findings confirm the transcription factor RhoprKr-h1 and the main YPP to be situated downstream of Met-Tai complex activation. Interestingly, the YPPs *RHBP* and *RhoprLp* are not affected to the same extent as is *RhoprVg* (Supplementary Figure S5A,B and Figure 3A,B). *RhoprKr-h1* (*p* < 0.05 for dsMet and *p =* 0.08 for dsTai, *n* = 5–6) and *RhoprVg* mRNA levels (*p* < 0.05, *n* = 5–6) in the ovaries are also negatively affected after down-regulation of *RhoprMet* or *RhoprTai* (Figure 3A’,B’ and Supplementary Figure S5A’,B’); however, *RhoprVgR* and *RhopLpR* transcripts do not show a similar pattern (Supplementary Figure S5A’,B’). Additionally, the total protein content in the fat body of dsRNA-treated insects (dsARG: 182.5 ± 8.5 µg/tissue; dsMet: 70.4 ± 26.1 µg/tissue; dsTai: 116.4 ± 11.5 µg/tissue) is lower when compared to controls (*p* < 0.01 for dsMet and *p* < 0.05 for dsTai, *n* = 5–6) (Figure 3C) and the total protein circulating in the hemolymph also decreases (dsARG: 29.13 ± 2.7 µg/µL of hemolymph; dsMet: 21.6 ± 1.7 µg/µL of hemolymph; dsTai: 23.29 ± 1.79 µg/µL of hemolymph; *p* < 0.05, *n* = 5–6) (Figure 3D,D’). This is mainly due to a decrease in the main YPP, Vg, in the hemolymph, as determined by ELISA (dsARG: 17.05 ± 2.8 µg/µL of hemolymph; dsMet: 4.6 ± 1.2; dsTai: 6.01 ± 1.7 µg/µL of hemolymph; *p* < 0.05, *n* = 5–6) and Western blot (Figure 3E,E’). Total protein content also significantly decreased in the ovaries of dsMet- and dsTai-injected insects (*p* < 0.0001, *n* = 5–6) (Figure 3F). It should be noted that the main lipoprotein in the insect hemolymph is Lp along with Vg [26]. Although RhoprLp mRNA levels are not severely affected, a drastic reduction of RhoprVg in dsRNA-injected insects indicated this Vg is responsible for the significantly reduced total circulating lipid (*p* < 0.05, *n* = 5–6) (Figure 3A,B and Supplementary Figures S5 and S6).

- Result of knockdown of *RhoprMet* and *RhoprTai* on the reproductive performance

**Figure 4:**
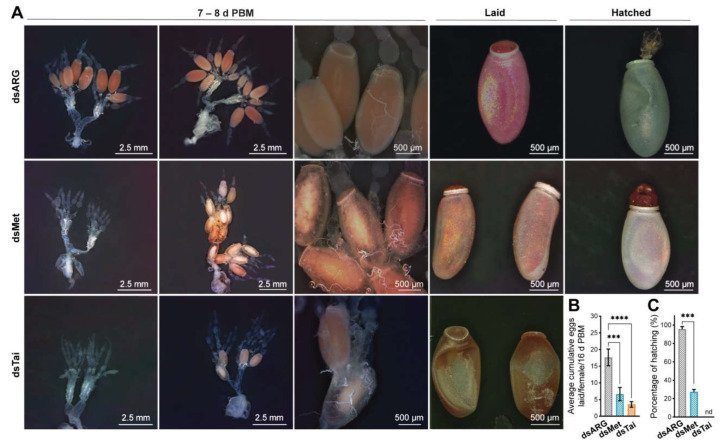
Reproductive fitness cost in RhoprMet and RhoprTai-deficient females. (**A**) (**left** panel): representative images showing the ovarian morphology of dsRNA-injected females at 7–8 days post blood meal (d PBM) ((**upper**), dsARG; (**middle**) dsMet; (**bottom**), dsTai). Note two phenotypes after each treatment: ovaries showing smaller basal follicles and no vitellogenic oocytes, or some eggs with less yolk content; (**right** panel): representative images showing the phenotype of eggs laid by RhoprMet and RhoprTai-injected females. Note smaller (dsMet) or with dented eggs (dsTai) (*n* = 15 to 20 females). (**B**) Total cumulative eggs laid per females throughout 16 d PBM (*n* = 15 to 20 females). *** *p* < 0.001; **** *p* < 0.0001 (One-way ANOVA and Tukey’s test as the post hoc test). (**C**) Percentage of hatching with respect to total eggs laid after each dsRNA treatment of 3 independent experiments. *** *p* < 0.001 (Student’s *t*-test); nd, not detected.

The overall effect of dsMet and dsTai injections on female reproduction was studied by examining ovarian and egg morphology, egg laying and hatchability. At 7–8 days post blood meal, two phenotypes are observed in knockdown females. After dsMet treatment, either basal oocyte growth is completely impaired, or oocyte growth begins but ultimately the yolk content inside the chorionated eggs is dramatically lower compared with controls as judged by phenotype (Figure 4A, middle panel). Arrested ovarian development became even more evident in dsTai-injected insects that also display the two phenotypes. The cumulative eggs laid per female throughout 16 days post blood meal was significantly lower in dsMet- and deTai-injected insects when compared to controls (dsARG: 17.6 ± 3; dsMet: 6.63 ± 3; dsTai: 3.5 ± 2, *n* = 15–20) (Figure 4B). Interestingly, the only eggs laid after dsTai injection are small and dented (Figure 4A, lower panel) and none of these hatched. The few eggs laid after dsMet injection show the same morphology as controls, but in general they are smaller (Figure 4A). Only ~20 % of the few eggs laid by dsMet-injected insects hatched (Figure 4C). Overall, transcript knockdown of *RhoprMet* and *RhoprTai* has a high reproductive cost.

- *RhoprMet* and *RhoprTai* appear to regulate JHSB_3_ biosynthesis in the corpus allatum

**Figure 5:**
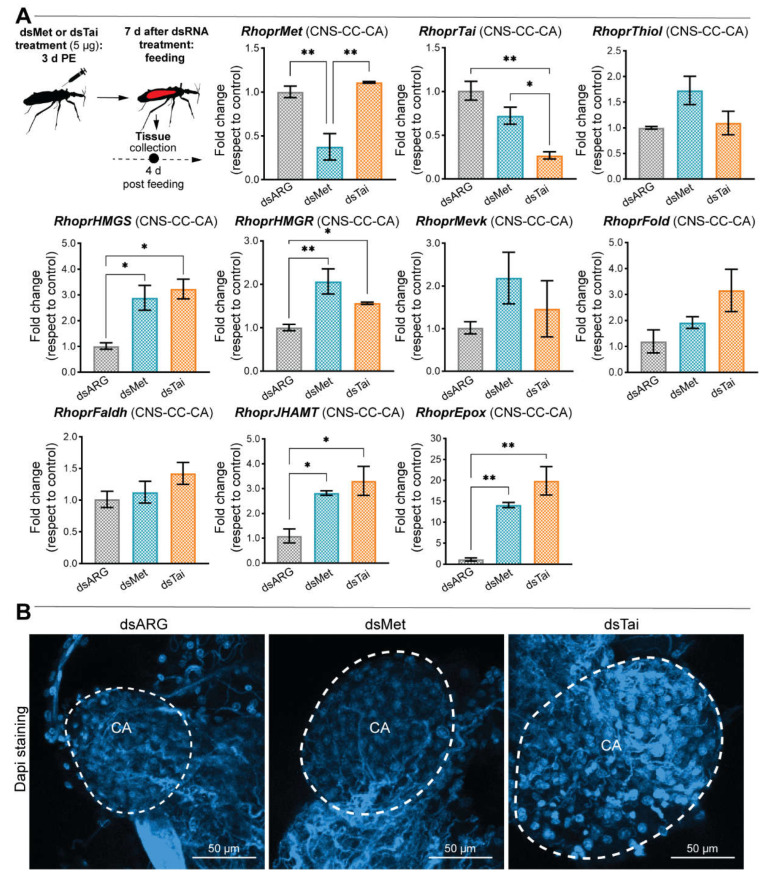
Effect of dsMet and dsTai injection on transcript levels of the JHSB_3_ biosynthetic enzymes 4 days post blood meal. (**A**) Upper left panel, experimental scheme. Transcript levels were quantified using RT-qPCR and analyzed by the 2^−ΔΔCt^ method. The y axes represent the fold change in expression relative to control (dsARG, value ~1) obtained via geometric averaging using *Rp49* and *actin* as reference genes. The results are shown as the mean ± SEM (*n* = 5–6, where each *n* represents a pool of CNS-CC-CA complexes from 2 insects). * *p* < 0.05; ** *p* < 0.01 (One-way ANOVA and Tukey’s test as the post hoc test). (**B**) CNS-CC-CA complex was dissected from dsRNA-injected insects 4 days PBM and mounted in Fluoroshield with DAPI (Sigma-Aldrich, ON, Canada). Image is representative of 3 tissues for each treatment.

The effects of knockdown of *RhopMet* and *RhoprTai* were also studied in the CNS—corpus cardiacum (CC)—CA complex. Although the major JH homologs described in insects have structural differences, they seem to be synthesized through a similar biosynthetic pathway, which includes 13 enzymatic steps, divided into early and late (JH-branch) steps [27]. The expression of transcripts for the JHSB_3_ biosynthetic enzymes was reported in *R. prolixus* [6]; indeed, high mRNA levels of *juvenile hormone acid methyltransferase* (RhoprJHAMT, known as the rate-limiting enzyme in JH biosynthesis) and of *methyl farneseoate epoxidase* (RhoprEpox, which catalyzes the final step to give the active hormone, JHSB_3_) overlapped with the major peak of JH titer, suggesting their relevance to JHSB_3_ production [6]. Here, we show that the transcript expression of two enzymes of the early steps, *HMG-CoA reductase* (RhoprHMGR) and *HMG-CoA synthase* (RhoprHMGS), is significantly up-regulated in dsMet- dsTai-injected insects (Figure 5A). In the late step, mRNA levels of *RhoprJHAMT* (*p* < 0.05, *n* = 5–6) and of *RhoprEpox* (*p* < 0.01, *n* = 5–6) are significantly up-regulated in dsMet- and dsTai-injected insects, the latter showing a drastic increase (20-fold) with respect to controls (Figure 5A). Accompanying this result, the size of the CA of dsMet- and deTai-injected females is larger with respect to controls (Figure 5B).

- RhoprKr-h1 does not appear to be involved in vitellogenesis but rather in successful hatching

**Figure 6:**
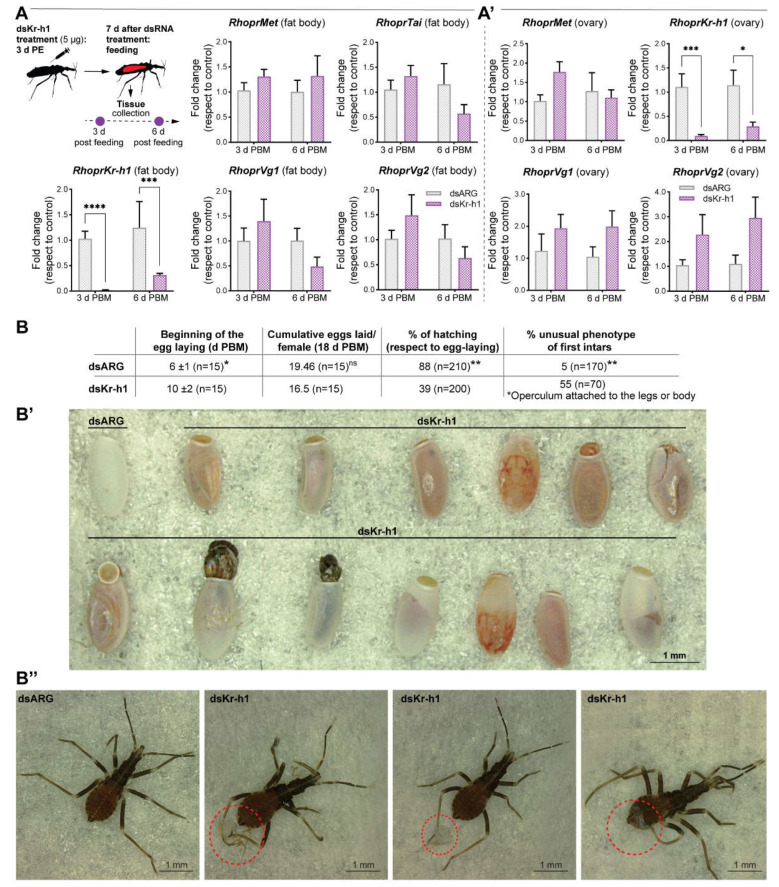
Effect of dsKr-h1 injection at different time points during vitellogenesis. (**A**,**A’**) *RhoprMet*, *RhoprTai*, *RhoprKr-h1* and *RhoprVg* mRNA expression in the fat body (**A**) and ovaries (**A’**) after dsKr-h1 injection. Transcript levels were quantified using RT-qPCR and analyzed by the 2^-ΔΔCt^ method. The y axes represent the fold change in expression relative to control (dsARG, value~1) obtained via geometric averaging using *Rp49* and *actin* as reference genes. The results are shown as the mean ± SEM (*n* = 5–6, where each *n* represents an individual tissue from 1 insect). * *p* < 0.05; *** *p* < 0.001; **** *p* < 0.0001 (Student’s *t*-test). (**B**) Parameters indicating reproductive fitness cost in dsKr-h1-injected females of 2 independent experiments. ** *p* < 0.01; ^ns^ *p* > 0.05 (Student’s *t*-test); ns, not significant (**B’**) Representative image showing the phenotype of eggs laid by control insects (**top left**) and dsKr-h1 injected females (**top right** and **bottom**). (**B’’**) Representative images showing the phenotype of first instars from eggs laid by dsKr-h1-injected females. Note the operculum attached to the legs or body.

Although RhoprKr-h1 is shown to be a JHSB_3_-response gene (Figure 2), its knockdown does not affect vitellogenesis, since the mRNA levels of the main YPPs in the fat body, *RhoprVg1*, *RhoprVg2* (Figure 6A), *RHBP* and *RhoprLp* (Supplementary Figure S7A), as well as of the endocytic receptors in the ovaries (Supplementary Figure S7A’), are not significantly different between dsKr-h1-injected insects and controls (*p* > 0.05, *n* = 5–6). Although there is a delay in the beginning of egg laying in dsKr-h1-injected females, no significant difference of the cumulative eggs laid per insect are observed with respect to control insects (dsARG: 19.46 eggs; dsKr-h1: 16.5 eggs, *n* = 15, *p* > 0.05) (Figure 6B). In addition, the eggs laid from all the treatments show no significant differences in time to hatch (not shown) but, interestingly, 39% of the eggs laid by females with *RhoprKr-h1* mRNA knockdown had severe morphological alterations (although some still appear to undergo embryogenesis) (Figure 6B’), and a decrease in hatchability (Figure 6B). Of the first instars that were able to hatch, 55% present an unusual phenotype, presumably due to issues during hatching, with parts of the operculum attached to the legs or to the body (Figure 6B’’).

## 3. Discussion

The JH signaling pathway is inherently complex, mainly because there is evident variability in the relative importance and function of downstream actions during insect reproduction, which appear to be related to the life cycle and life history strategies of individual insect species. Over the last nearly 100 years, most work on JH references the groundbreaking studies of Sir Vincent Wiglesworth on *R. prolixus* and JH [1,2], which were updated and expanded in the mid-nineties by Kenneth Davey [3,4]. More than 20 years after this, Villalobos-Sambucaro et al. [6] finally revealed the specific JH homolog responsible for the original observations of Wigglesworth and Davey in *R. prolixus*, to be JHSB_3_. One of the major contributions of our work is to unmask the JHSB_3_ signaling in *R. prolixus* reproductive physiology, and to discover new avenues for novel investigations in this field.

- JHSB_3_ stimulates yolk protein synthesis and negatively modulates endocytic receptor transcript expression

In *R. prolixus*, YPPs are primarily synthesized by the fat body during vitellogenesis. Once circulating in the hemolymph, YPPs are taken up by developing oocytes to be packaged into eggs. Although Vg is the main YPP, Lp (the main lipoprotein in insects) and RHBP (which generates the characteristic pink coloration of *R. prolixus* eggs) are also described as YPPs of critical importance [28]. There is strong evidence that, in addition to the fat body, the ovary also produces yolk proteins [22,28]. Here, we report that in the fat body of *R. prolixus*, *RhoprLp*, *RHBP* and the two Vg genes, *RhoprVg1* and *RhopVg2* are positively regulated after a blood meal (when JHSB_3_ titers are supposed to be increasing), and by direct hormone stimulation. It is noteworthy that the stronger JHSB_3_ response is for *RhoprVg*, which makes sense as vitellin (the form in which Vg is deposited into oocytes) represents between 60 to 90% of the total protein content of mature oocytes [28]. Villalobos-Sambucaro et al. [21], also suggested that in *R. prolixus,* the synthesis of the yolk protein RHBP and/or incorporation into the eggs could be controlled by JH. As expected, we also demonstrated that JHSB_3_ regulates *RhoprVg* expression via *RhoprMet* and *RhoprTai*. Further studies are needed to identify the JHSB_3_ binding site in RhoprMet, although in a close triatomine species using modelling and ligand docking studies, conserved critical amino acids in the PAS-B domain of Met were reported that resembled a JH-ligand pocket [29].

Melo et al. [22] reported that in large follicles of *R. prolixus* females, the uptake of Vg synthesized by the fat body is reduced, and so the Vg accumulated during the final phase of vitellogenesis is primarily ovarian Vg. Here, we report for the first time that the synthesis of *RhoprVg* by the ovaries is also under JHSB_3_ control, via Met-Tai complex, and this occurs mainly one day before choriogenesis begins.

RhoprVgR and RhoprLpR belong to the low-density lipoprotein receptor gene superfamily and play critical roles in oocyte development by mediating endocytosis of Vg and Lp, respectively [30]. Here, we demonstrate that at the beginning of vitellogenesis, *RhoprVgR* and *RhoprLpR* transcript expression in ovaries decrease. Interestingly, JHSB_3_ treatment by both in vivo or ex vivo assays, promotes a significant down-regulation of the endocytic receptors, thus confirming that JHSB_3_ is negatively modulating their expression. However, knockdown of *RhoprMet* or *RhoprTai* shows a weak impact on the endocytic receptor levels in the ovaries. These results suggest that the control of JHSB_3_ on RhoprVgR and RhoprLpR could be not via the Met-Tai complex but rather via other signaling. For example, in *Locusta migratoria*, JH promotes VgR phosphorylation for intracellular recycling through a JH-membrane receptor [31]. Thus, in *R. prolixus*, JHSB_3_ seems to be inhibiting the transcript expression of endocytic receptors and, perhaps, modulating protein recycling.

- Both RhoprMet and RhoprTai are critically important for successful vitellogenesis

Nuclear receptor regulation is an important mechanism for coordinating hormone action in a temporal and tissue-specific manner [32]. The differential quantitative expression observed in different tissues could play an important role in determining effects of the ligand, in this case, namely JHSB_3_. The higher *RhoprMet* and *RhoprTai* mRNA expression in the CNS, the fat body and ovaries can determine its hormone responsiveness; but the expression may change during a specific physiological state. In *R. prolixus*, the transcript expression of both nuclear factors is not particularly regulated by the blood meal. In fact, JHSB_3_ topically applied to adult females shows no effect on *RhoprMet* or *RhoprTai* expression in the fat body, and just a slight decline in *RhoprMet* levels in the ovaries. A similar profile was reported for Met in adult females of the desert locust, *Schistocerca gregaria* [33], the cockroaches, *Diploptera punctata* and *Blattella germanica* [34,35], the cotton bollworm, *Helicoverpa armigera*, and in *T. castaneum* [36,37]. In contrast, in adult *A. aegypti* JH stimulates the production of two Tai isoforms in the fat body while reducing the levels of two others [38]. Overall, the data presented here indicate that *RhoprMet* and *RhoprTai* are not JH- or nutritional-response genes. However, when we assayed exogenous JHSB_3_ by ex vivo experiments, the levels of both transcription factors were increased. The JH receptor Met resembles in action the vertebrate Aryl hydrocarbon receptor (AhR) [39]. An up- or down-regulation of AhR levels occurs in challenges with the same ligands but at different concentrations or time periods [40]. Here, by ex vivo experiments, only the fat body or the ovaries were exposed to the hormone, and the data suggest that *RhoprMet* and *RhoprTai* mRNA levels might be directly regulated in these tissues by the presence of JHSB_3_.

To test whether RhoprMet and RhoprTai individually are required for a successful gonadotropic cycle in *R. prolixus*, RNAi-mediated knockdown of these transcripts was performed. After the blood meal, when vitellogenesis begins, dsMet- and dsTai-injected insects were unable to complete a reproductive cycle. While there are robust data available showing that Met plays a role in transducing the JH signal in insect reproduction, the possible role of Tai in this process has been less studied. In the bugs *Pyrrhocoris apterus* and *C. lectularius,* and in *L. migratoria*, the silencing of Met or Tai blocks ovarian development and suppresses Vg gene expression [41,42]. Interestingly, Tai is not only involved in JH signaling but also in the ecdysteroid pathway; in *D. melanogaster*, *A. aegypti* and *B. germanica*, Tai modulates ecdysteroid signaling [43,44,45]. In *R. prolixus*, ecdysteroid signaling has a critical role in reproductive physiology, e.g., a key signal for oviposition [23]. Here, we show that the negative impact after *RhoprTai* silencing for successful reproduction is stronger than that observed after *RhoprMet* knockdown, which might suggest that in dsTai-injected insects, reproduction is doubly affected; thus, RhoprTai could be involved in crosstalk between JH and ecdysteroid signaling.

- RhoprMet and RhoprTai appear to regulate JHSB_3_ biosynthesis in the corpus allatum

The impact of *RhoprMet* and *RhoprTai* transcript knockdown was also studied in the CNS-CC-CA complex, since the CA is the main site of JH biosynthesis in *R. prolixus* [6]. The JHAMT enzyme has been proposed as the rate-limiting enzyme in JH biosynthesis, and its mRNA level and enzyme activity are closely correlated with the patterns of JH synthesis [6,46]. In addition, the Epox enzyme seems to be important for improving the agonistic activity of the Met receptor; indeed, epoxidated JH confers reproductive advantages in mosquitos [47]. Here, we introduce a possible new form of JH regulation in *R. prolixus* whereby the Met-Tai complex might act as a hormonal sensor in the CA, promoting a negative feedback loop that keeps JHSB_3_ biosynthesis and CA activity under control; a homeostatic mechanism also suggested in *S. gregaria* and *P. apterus* [33,48]. In *B. germanica*, there is a correlation between the CA size and JH biosynthesis [49]. Here, we show that the CA increases in size after *RhoprMet* and *RhoprTai* silencing, and so hypothesize that in *R. prolixus*, the CA size also correlates with its biosynthetic activity. Now that part of the mechanism and target genes underlying JH action in *R. prolixus* reproduction are more clearly understood, further analysis (e.g., silencing key enzymes of the JHSB_3_ biosynthetic pathway) should unmask if JHSB_3_ precursors have similar or different effects on *R. prolixus* reproduction.

- RhoprKr-h1 does not appear to be involved in vitellogenesis but rather in successful hatching

According to the current model of the JH signaling pathway, the transcription factor Kr-h1 is expressed and functions downstream of JH-Met activation. Here, we report that the blood meal and exogenous JHSB_3_ treatment cause a rapid and large increase in *RhoprKr-h1* expression in both the fat body and ovaries. The expression of JH-regulated genes, such as Kr-h1, is a reliable indicator for JH titers [46]. The titer of JHSB_3_ after a blood meal in adult female *R. prolixus* has not yet been reported. Here, RhoprKr-h1 mRNA levels are minimally 10-fold higher at all the time points analyzed after feeding relative to unfed insects, suggesting the expected increase of JHSB_3_ titers induced by the blood meal. However, a role of RhoprKr-h1 during vitellogenesis is not evident. In *A. aegypti*, JH activates Kr-h1 expression through a hypothetical cell membrane-associated JH receptor via phospholipase C signaling. This signaling phosphorylates Met and Tai by a calcium/calmodulin-dependent protein kinase II (CaMKII), which then positively regulates their binding with JHRE, thereby activating target gene expression, such as Kr-h1 [50]. In *R. prolixus*, the suppression of the Met-Tai complex by RNAi decreases *RhoprKr-h1* transcript levels in the fat body and ovaries. However, it remains to be elucidated whether a cell membrane-based JHSB_3_ receptor, as in *A. aegypti*, is regulating *RhoprKr-h1* expression, or if JHSB_3_ is acting directly on its nuclear receptor Met. The role of Kr-h1 in female reproduction varies across species; in most of the insects studied, *Kr-h1* transcript knockdown leads to a reduction of Vg and impaired oocyte maturation [51]. However, in the hemipteran *P. apterus* and *C. lectularius*, Kr-h1 depletion neither reduces Vg expression nor affects ovarian development, whereas JH deprivation or knockdown of Met or Tai mRNA do [41,42]. We demonstrated that *RhopKr-h1* transcript knockdown females can lay eggs at the same rate as control insects but only 40% of the eggs hatched and a high percentage of first instars showed an unusual phenotype. Two features emerge from these results. Because insect oocytes are transcriptionally silent during vitellogenesis [52], the potential RhoprKr-h1 content in the eggs could derive from a maternal origin. When it is down-regulated in adult females, the content in the oocytes should also decrease, negatively affecting the potential regulation that this factor could have during the early oogenesis and/or embryogenesis. However, it remains to be elucidated how *RhoprKr-h1* could be incorporated into the eggs, although a likely source would be the nurse cells that are known to transfer transcripts into the oocyte via the nutritive cords [53]. In addition, maternal dsKr-h1 could potentially accumulate in oocytes and affect embryo development. In *B. germanica* the parental down-regulation of Kr-h1 mRNA via RNAi results in morphological malformations of embryos [54].

Some *RhoprKr-h1* mRNA knockdown eggs have also defects associated with hatching but still appear to undergo embryogenesis. Components of the eggshell are synthesized and secreted by follicle cells surrounding the maturing oocyte. *RhoprKr-h1* mRNA knockdown might alter the regular composition of the chorion and thus impair hatchability, as suggested in *B. germanica* [54].

## 4. Materials and Methods

- Experimental Animals

*R. prolixus* were obtained from an established colony at the University of Toronto Mississauga. Insects were reared in incubators at 25 °C under high humidity (~50%) and artificially fed as detailed by Orchard et al. [55]. Adult mated females for experiments were obtained as previously described [56]. Briefly, males and females were separated during the last nymphal instar and at 30 days post-ecdysis were fed on defibrinated rabbit blood (Cedarlane Laboratories Inc., Burlington, ON, Canada). Insects that gorged at least nine times their own initial body weight were chosen and allowed to ecdyse into adults. Newly emerged adult females were segregated individually and placed together with a recently fed male. Mating was verified by examining the cubicle for the deposited spermatophore. After copulation, if required, mated females 10 d post-ecdysis to adult were fed and only those that gorged 2.5 to 3 times their initial body weight were used for experiments.

- Phylogenetic Analysis of RhoprTai and RhoprKr-h1

To confirm the identities of RhoprTai and RhoprKr-h1 sequences, the evolutionary history, conducted in MEGA X [57], was inferred by using the Maximum Likelihood method and frequency-response model [58]. The bootstrap consensus trees inferred from 1000 replicates is taken to represent the evolutionary history of the taxa analyzed. Initial trees for the heuristic search were obtained automatically by applying the Maximum Parsimony method. A discrete Gamma distribution was used to model evolutionary rate differences among sites. The analysis involved 22 (for RhoprKr-h1) and 23 (for RhoprTai) amino acid sequences. All positions with less than 95% site coverage were eliminated.

- RNA extraction and reverse transcription/quantitative PCR (RT-qPCR)

Total RNA was extracted from tissues of adult females *R. prolixus* using TRIzol reagent (Invitrogen by Thermo Fisher Scientific, Waltham, MA, USA) according to the manufacturer’s instructions. First-strand complementary DNA (cDNA) synthesis using 1 µg total RNA and qRT-PCR assays were performed as previously described [56]. *Actin*, *Rp49* (*60S ribosomal protein L32*) or *18S ribosomal RNA* (*18S rRNA*) housekeeping genes were used as reference genes to normalize the target gene expression (Table S1). For each pair of primers, the efficiency ranged from 85 to 110%, with linear correlation coefficients (r^2^) ranging from 0.8 to 1, and the dissociation curves always showed a single peak. The tissue distribution and temporal mRNA expression were calculated relative to 1000 copies of the average of the reference genes using 2^−ΔCt^ method [59]. For the rest of the experiments, the results are shown as fold change quantified relative to the expression of control samples, using 2^−ΔΔCt^ method following of the geometric mean of the reference genes [60]. The results are shown as the mean ± SEM of *n* = 4–5, where each *n* represents a pool of tissues from 3 insects. Experiments were repeated with two technical replicates and using no-template controls.

- JHSB_3_ treatment: in vivo experiments

Newly emerged adult females were topically treated on the cuticle of the dorsal abdomen with either 50 pg of JHSB_3_ (Toronto Research Chemical, North York, ON, Canada) dissolved in 10 μL of acetone, or with 10 μL of acetone (controls) [61]. The fat body, ovaries and hemolymph were individually sampled 6 and 24 h later and processed for RT-qPCR or ELISA. The results are shown as the mean ± SEM of *n* = 5–6, where each *n* represents a tissue from 1 insect.

- JHSB_3_ treatment: ex vivo experiments

Incubation of the fat body or ovaries was carried out in glass silanized vials (Thermo Fisher Scientific, Waltham, MA, USA), using 200 μL of Grace’s medium, with L-glutamine and without insect hemolymph (Millipore-Sigma, Oakville, ON, Canada) as earlier described [62,63]. JHSB_3_ was dissolved in acetone and added to the medium at a final concentration of 35 nM; controls were incubated with the same volume of the corresponding solvent alone. The fat body, ovaries and incubation medium were individually collected after 4 h of incubation (in the dark, at 28 °C and with gentle shaking) and processed for RT-qPCR or ELISA. The results are shown as the mean ± SEM of *n* = 5–6, where each *n* represents a tissue from 1 insect.

- RNAi assay

To knock down *RhoprMet*, *RhoprTai* and *RhoprKr-h1* transcripts, two non-overlapping fragments were generated. Preliminary RNAi experiments yielded the same ovarian phenotype using each fragment individually, minimizing the probability of off-target effects. To increase the efficiency of the down-regulation, we next injected both constructs together to conduct the studies. Double-Stranded RNA (dsRNA) molecules were synthesized using the T7 Ribomax Express RNAi System (Promega, WI, USA), according to the manufacturer’s protocol. Gene specific primers are shown in Supplementary Table S1. As an experimental control, a dsRNA molecule encoding the partial sequence of ampicillin resistance gene (dsARG) was obtained from the pGEM-T Easy Vector system (Promega, WI, USA) and used throughout the study.

The number of Tai isoforms in *R. prolixus* has not been elucidated. In the genome, a single gene can be identified. Between 2–4 isoforms of Tai have been reported in insects due to the splicing variants at the 3′ [45]. The primers for dsRNA synthesis were designed in the middle of the sequence, so all potential isoforms were included. The knock-down of *RhoprMet* was also done considering a part of the sequence shared between both isoforms, RhoprMet1 and RhoprMet2.

To knock-down the expression of *RhoprMet*, *RhoprTai* and *RhoprKr-h1*, females 3 days post-ecdysis into adult were separated into four groups. The females of each group were injected into the hemocoel with 5 μg of each dsRNA diluted in 5 μL of ultrapure water using a Hamilton micro syringe. Seven days after injection, the females were given a blood meal and placed together with a recently fed male for mating. The potential injury of the injection or its inherent physiological effect, did not affect the feeding behavior, all groups of injected insects had comparable weight before and after feeding. At 3, 4 and 6 days post-injection, the fat body, ovaries, CNS and hemolymph were collected and processed for RT-qPCR (*n* = 5–6, where each *n* represents a tissue from 1 insect), ELISA (*n* = 5–6, where each *n* represents hemolymph from 1 insect), Western blot (*n* = 3, where each *n* represents hemolymph from 1 insect) or lipid and protein determination, as indicated. Other groups were used to examine ovarian morphology (*n* = 15–20) (photographed with a digital microscope (Leica DVM6, Wetzlar, Germany)), egg laying (*n* = 15–20) and hatchability according to Leyria et al. [56].

- Protein and lipid measurements

For protein determination, the fat body and ovaries were treated as described by Leyria et al. [64]. Hemolymph samples were obtained using a micro syringe from cut ends of the legs and collected as described by Leyria et al. [63]. Protein quantification was done by BCA protein quantification assay (Pierce™ BCA Protein Assay Kit, Thermo Fisher, Mississauga, ON, Canada). The results are shown as the mean ± SEM of *n* = 5–6, where each *n* represents a tissue or hemolymph from 1 insect.

For lipid determination, 5 μL of hemolymph was collected and immediately placed in 50 μL of 10 % trichloroacetic acid and centrifuged for 5 min at 20 °C, 8000× *g*. Pellets containing lipids associated with lipoproteins were resuspended in 200 μL isopropanol and used for lipid level measurements using a colorimetric assay, as previously detailed [64]. The results are shown as the mean ± SEM of *n* = 5–6, where each *n* represents hemolymph from 1 insect.

- SDS-PAGE and Western blot

Protein bands from 1 μL hemolymph of dsRNA-treated insects were separated under reducing conditions on pre-made gels (percentage 4–20 %, Mini-Protean TGX Stain-Free Precast Gels, BioRad, Mississauga, ON, Canada). The gel was then stained with QC Colloidal Coomassie (BioRad), for 1 h at room temperature with gentle shaking. After washing, the gel was imaged on a ChemiDoc XRS system (BioRad). To Western blot, 1 μL of hemolymph (1:20 dilution) for each dsRNA treatment was used to separate protein under reducing conditions on a pre-made gels. A detailed protocol to Western blot was previously described (56). The polyclonal anti-Vg antibody was commercially acquired from Boster Biological Technology (Pleasanton, CA, USA), using as antigen a shared portion of the RhoprVg sequences (gene accession numbers: RPRC002109 and RPRC013511; recombinant antigen sequence: PLPQFVLQSRPELVPLPKLVAGGQVLDIVKTKNYSNCEQRMAYHFGLTGLTDWEPASNQ). Primary antibody (dilution 1:2000) was incubated overnight at 4 °C with gentle shaking. Horseradish peroxidase (HPR) conjugated goat anti-rabbit IgG (secondary antibody, dilution 1:5000) was incubated for 1 h at room temperature. Blots were visualized using enhanced chemiluminescence (Clarity Western ECL Substrate, BioRad), imaged on a ChemiDoc XRS system and analyzed using Image Lab 5.0 (BioRad Software and System). A pre-adsorption (“blocking”) control was performed in order to evaluate the specificity of the signal obtained. The anti-Vg antibody was pre-incubated overnight at 4 °C with a molar excess of the immunogen (final concentration: 1 ng/µL) and then used for Western blot using fat body tissues (Supplementary Figure S8). The images shown are representatives of 3 independent experiments.

- Quantification of vitellogenin by ELISA

Quantification of Vg in the hemolymph or released by the fat body or ovaries after ex vivo incubation, was carried out by the enzyme-linked immunosorbent assay (ELISA) as descripted by Aguirre et al. [65]. Microtiter plates were loaded with two technical replicates using 200 μL/well of standard Vg or with appropriate hemolymph dilutions in buffer carbonate (15 mM Na_2_CO_3_, 35 mM NaHCO_3_, pH 9.6) or aliquots of medium, as indicated, and incubated for 90 min at 37 °C. Plates were washed four times with phosphate-buffered saline-Tween (PBST: 8.2 mM Na_2_HPO_4_, 1.5 mM KH_2_PO_4_, 150 mM NaCl, 2.7 mM KCl, 0.05% Tween 20, pH 7.4) and incubated with anti-Vg antibody (0.01 µg/mL, 60 min at 37 °C) in PBST containing 0.1% of bovine serum albumin. Plates were then washed as described above and loaded with anti-rabbit immunoglobulin conjugated to HPR in PBST (1:5000; 30 min at 37 °C). After washing, plates were incubated with TMB Liquid Substrate System (Millipore-Sigma, Oakville, ON, Canada). The enzyme reaction was allowed to proceed for 15 min and then stopped with 4 N H_2_SO_4_. Plates were read at 492 nm using a multi-mode reader (Synergy HTX from Agilent Technologies, Santa Clara, CA, USA). A standard curve using the recombinant antigen sequence (from 50 pg/mL to 10 mg/mL) was performed in parallel. The results are shown as the mean ± SEM of *n* = 5–6, where each n represents hemolymph from 1 insect. The experiment was performed twice.

- Statistical Analyses

All data were processed using the GraphPad Prism Software (GraphPad Software, San Diego, CA, USA). All datasets passed normality and homoscedasticity tests. Significance of differences were determined either with one-tailed Student’s t test, or with one-way ANOVA followed by Tukey’s test, as indicated.

## 5. Conclusions

**Figure 7:**
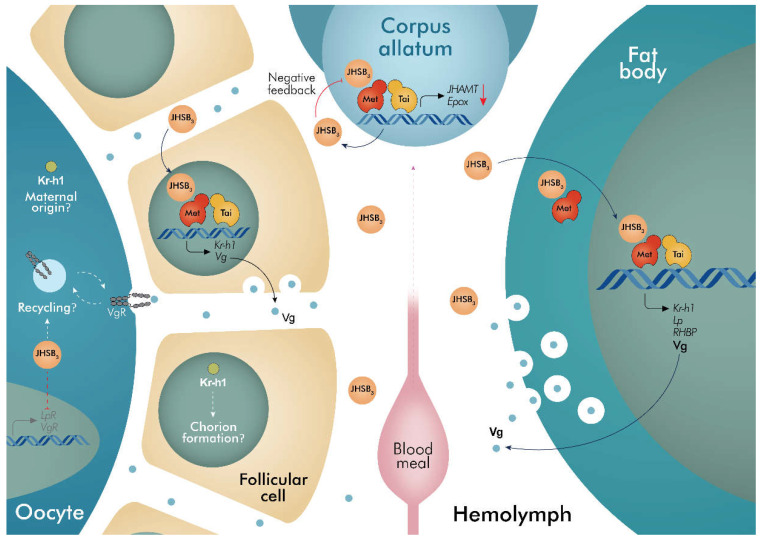
Insights on the possible mode of action of JHSB_3_ in egg production in *R. prolixus* females. After a blood meal, JHSB_3_ regulates expression of yolk protein precursors (YPPs) through Met-Tai complex activation, mainly in the fat body but also in the ovarian follicles, the second YPPs source. It remains to be elucidated if this stimulation is due to Met working directly on the promoters of the YPPs. *RhoprKr-h1* transcript expression is also highly dependent on JHSB_3_ signaling; however, it does not appear to be involved in vitellogenesis. In the model, we suggest that Kr-h1 may accumulate in the eggs from a maternal source and impact late embryogenesis as well as possibly having implications in chorion formation by the follicular cells and thereby successful hatching. In the CA, the Met-Tai complex might act as a JH sensor through a negative feedback loop, directly modulating the expression of biosynthetic enzymes. In the ovary, JHSB_3_ inhibits the transcript expression of the endocytic receptors *VgR* and *LpR*, probably promoting protein recycling. Together, these coordinated actions lead to a successful reproductive cycle. JHSB_3_, JH III skipped bisepoxide; Met, Methoprene tolerant; Tai, Taiman; Kr-h1, Krüppel homolog 1; Vg, vitellogenin; Lp, lipophorin; VgR, vitellogenin receptor; RHBP, Rhodnius heme binding protein; JHAMT, Juvenile hormone acid methyltransferase; Epox, Methyl farneseoate epoxidase.

Mechanistic understanding of transcript regulation of each component of the JHSB_3_-Met-Tai axis is critical to reveal their complete role in *R. prolixus* reproductive physiology. Collectively, the data presented here unmask crucial molecular parts of the reproductive physiology in this classic model and proposes several novel avenues for further exploration. Considering the literature already reported in other insects and taking into account the results presented here, a proposed schematic model for the role of JHSB_3_ transducing signals that mediate successful reproduction is shown in Figure 7. After a blood meal, JHSB_3_ is synthesized and released from the CA into the hemolymph. In the CA, Met-Tai complex might act as a JH sensor through a negative feedback loop, directly modulating the transcription of biosynthetic enzymes to keep JHSB_3_ levels and CA activity under control. Once circulating in the hemolymph, JHSB_3_ mediates a coordinated action on two tissues, the fat body and ovaries, mainly regulating the massive production of YPPs, with the YPPs taken up by developing oocytes. These JHSB_3_ functions are accomplished through the Met-Tai complex. In the ovaries, JHSB_3_ inhibits transcript expression of the endocytic receptors VgR and LpR (probably promoting protein recycling) but this does not seem be through the classic JH-Met-Tai axis. Whether Met works directly on the promoters of target genes remains to be elucidated. Although Met-Tai activation induces *Kr-h1* transcript expression in both the fat body and the ovaries, its role in vitellogenesis was not evident. However, Kr-h1 might possibly accumulate in the eggs as a maternal source affecting late embryogenesis, as well as having implications in chorion formation by the follicular cells, and thus its silencing could impair successful development or hatchability. Taken together, these coordinated actions of JHSB_3_ signaling lead to successful reproduction.

## Data Availability

The data that supports the findings of this study are available in the main text and supplementary materials of this article.

## Supplementary Materials

The following supporting information can be downloaded at: https://www.mdpi.com/article/10.3390/ijms232213832/s1.

## Abbreviations

JHs, Juvenile hormones; CA, corpus allatum; JHSB3, JH III skipped bisepoxide; Vg, Vitellogenin; Met, Methoprene-tolerant; bHLH, basic-helix-loop-helix; JHRE, JH response elements; Kr-h1, Zinc-finger transcription factor Krüppel homolog 1; Tai, Taiman; YPPs, yolk protein precursors; RNAi, RNA interference; CNS, central nervous system; RHBP, Rhodnius heme binding protein; Lp, lipophorin; LpR, lipophorin receptor; VgR, vitellogenin receptor; ELISA, enzyme-linked immunosorbent assay; ARG, ampicillin resistance gene; AhR, Aryl hydrocarbon receptor; CC, corpus cardiacum; CaMKII, calcium/calmodulin-dependent protein kinase II; qRT-PCR, RNA extraction and reverse transcription/quantitative PCR; PBST, phosphate-buffered saline-Tween; HPR, horseradish peroxidase; Actin, β-actin; Rp49, 60S ribosomal protein L32; 18S, 18S ribosomal RNA; Acetyl-CoA-thiolase; HMGR, HMG-CoA reductase; HMGS, HMG-CoA synthase; MEVK, Mevalonate Kinase; FOLD, Farnesol dehydrogenase; FALDH, Farnesol dehydrogenase; JHAMT, Juvenile hormone acid methyltransferase; Epox, Methyl farneseoate epoxidase; ds, double stranded; SRC, steroid receptor coactivator family proteins; NRC, nuclear receptor coactivator; FISC, βFtz-F1 Interacting Steroid receptor Coactivator.
