# Supplementary material for: Impact of JH Signaling on Reproductive Physiology of the Classical Insect Model, Rhodnius prolixus
Source: Int J Mol Sci. 2022 Nov 10;23(22):13832. doi: 10.3390/ijms232213832 (PMC9692686; doi:10.3390/ijms232213832)
Supplement: Supplementary file 1 [file ijms-23-13832-s001.zip › ijms-1996375-supplementary.pdf]

Supplementary

Impact of JH Signaling on Reproductive Physiology of the Classical Insect Model, *Rhodnius prolixus*

Jimena Leyria *, Ian Orchard and Angela B. Lange

Department of Biology, University of Toronto Mississauga, Mississauga, ON, L5L 1C6, Canada
*Correspondence: jimenal.leyria@utoronto.ca; Tel.: +1-(905)-569-4752

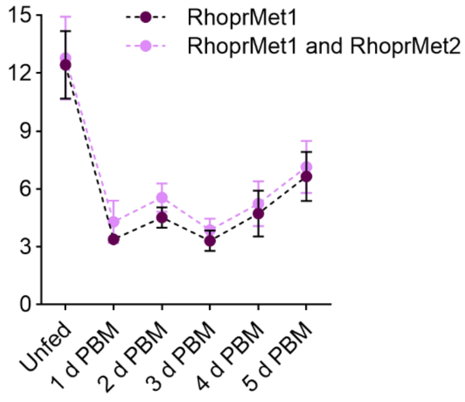

Supplementary Figure S1. ***RhoprMet1*** **and** ***total RhoprMet*** **(RhoprMet1 + RhoprMet2) transcript expression.** Expression profiles of the Met isoforms in the fat body of adult females were examined by RTPCR at 10 d post ecdysis (unfed) and throughout 5 days post blood meal (d PBM). The transcript expression was quantified using RT-qPCR and analyzed by the $2^{-\Delta Ct}$ method. The y axis represents the relative expression obtained via geometric averaging using *Rp49*, *18S rRNA* and *actin* as reference genes. The results are shown as the mean ± SEM (n = 5, where each n represents a pool of tissues from 3 insects). No significant differences were found between *RhoprMe1* and *total RhoprMet* transcript expression (Two-way ANOVA and a Tukey's test as the post hoc test).

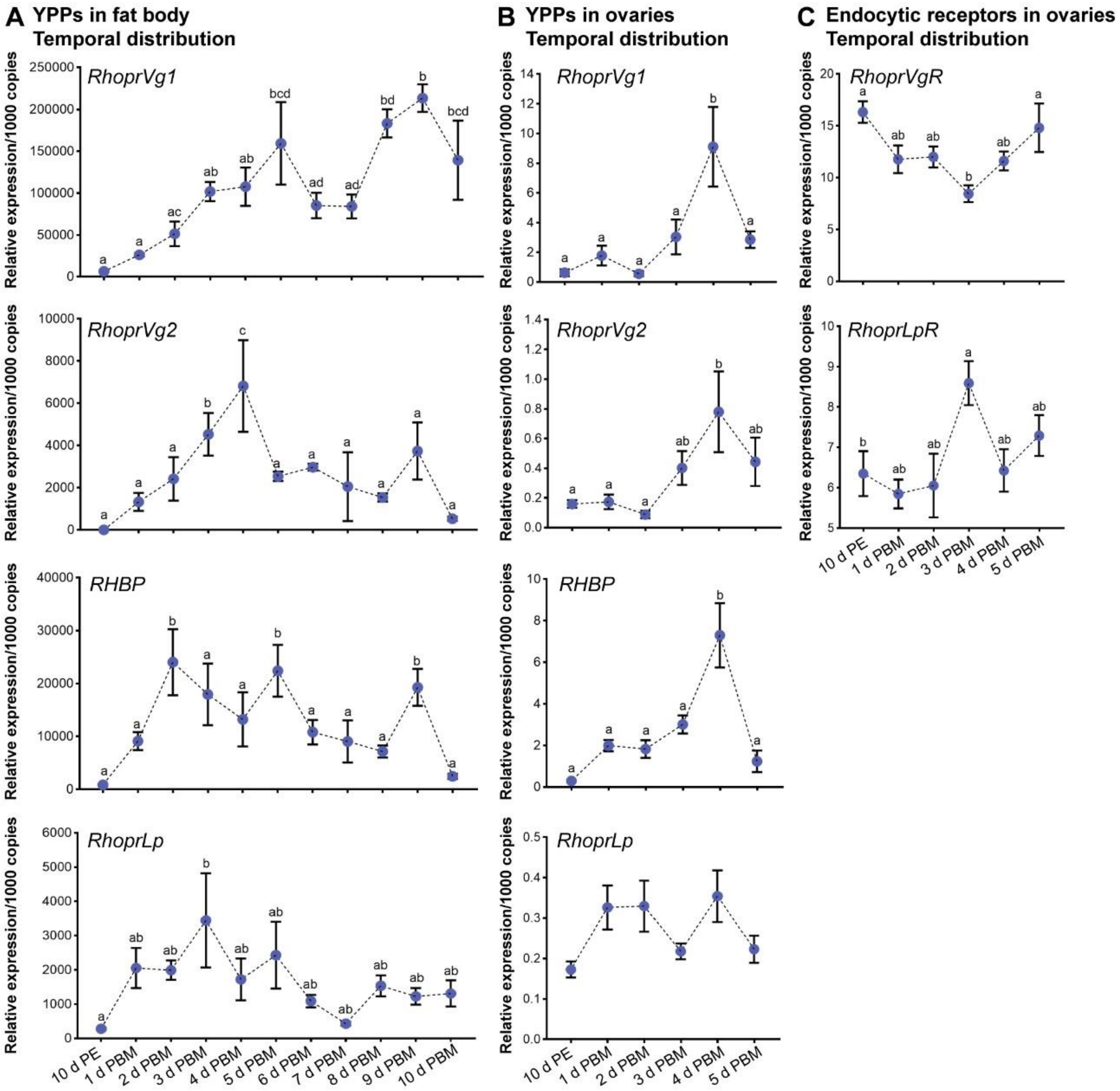

Supplementary Figure S2. Temporal transcript expression of yolk protein precursors (YPPs) and endocytic receptors. **(A)** *RhoprVg1*, *RhoprVg2*, *RHBP* and *RhoprLp* transcript levels in the fat body at 10 d post ecdysis (d PE) and throughout 10 days post blood meal (d PBM); **(B)** *RhoprVg1*, *RhoprVg2*, *RHBP* and *RhoprLp* transcript levels in the ovaries at 10 d PE and throughout 5 d PBM (prior to oviposition); **(C)** *RhoprLpR* and *RhoprVgR* transcript expression in the ovaries at 10 d PE and throughout 5 d PBM. The transcript levels were quantified using RT-qPCR and analyzed by the $2^{-\Delta Ct}$ method. The y axes represent the relative expression obtained via geometric averaging using *Rp49, 18S rRNA* and *actin* as reference genes. The results are shown as the mean ± SEM (n = 4-5, where each n represents a pool of tissues from 3 insects). Different letters indicate significant differences at $p<0.05$ (One-way ANOVA and Tukey's test as the post hoc test).

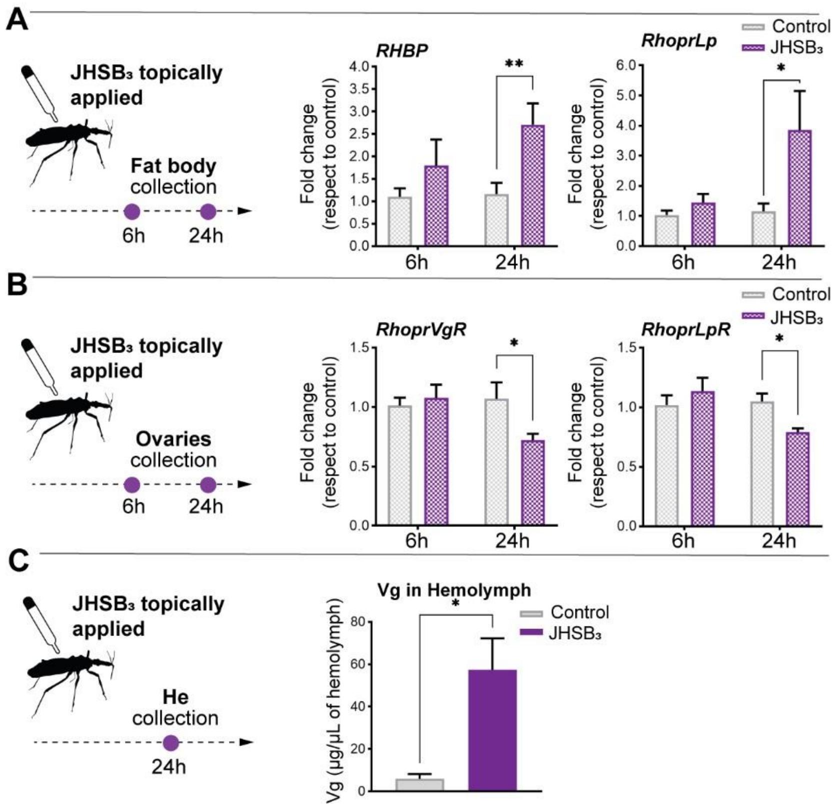

Supplementary Figure S3. In vivo assays: effect of JHSB3 treatment (50 pg in 10 µL of acetone) on mRNA expression of yolk protein precursors and endocytic receptors in the fat body and ovaries, and vitellogenin levels in the hemolymph. JHSB3 was topically applied in newly emerged adult females, and transcript levels of *RHBP* and *RhoprLp* in the fat body **(A)**, and *RhoprVgR* and *RhoprLpR* in the ovaries **(B)** were measured at 6 and 24 h after topical application. Transcript expression was quantified using RT-qPCR and analyzed using the $2^{-\Delta\Delta Ct}$ method. The y axes represent fold change in expression relative to control (10 µL of acetone, value ~ 1) obtained via geometric averaging using *Rp49* and *actin* as reference genes. The results are shown as the mean ± SEM (n = 5, where each n represents an individual tissue from 1 insect). (C) Vitellogenin (Vg) levels in the hemolymph (He) were measured by ELISA at 24 h after the treatment. The results are shown as the mean ± SEM (n = 5, where each n represents the hemolymph from 1 insect). *$p<0.05$; **$p<0.01$ (Student's t-test).

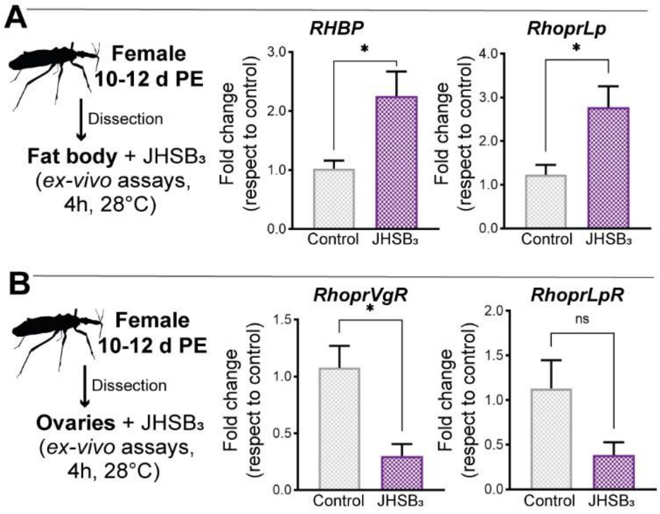

Supplementary Figure S4. Ex vivo assays: effect of JHSB$_3$ treatment (35 nM) on mRNA expression of yolk protein precursors and endocytic receptors in the fat body and ovaries. JHSB$_3$ was dissolved in acetone, and then added to the incubation medium containing an individual fat body or ovary, as indicated. Transcript levels of *RHBP* and *RhoprLp* in the fat body **(A)**, and *RhoprVgR* and *RhoprLpR* in the ovaries **(B)**, were quantified using RT-qPCR and analyzed by the $2^{-\Delta\Delta Ct}$ method. The y axes represent fold change in expression relative to control (acetone, value ~ 1) obtained via geometric averaging using *Rp49* and *actin* as reference genes. The results are shown as the mean ± SEM (n = 5, where each n represents an individual tissue from 1 insect). *p<0.05; ns, non-significant (Student's t-test).

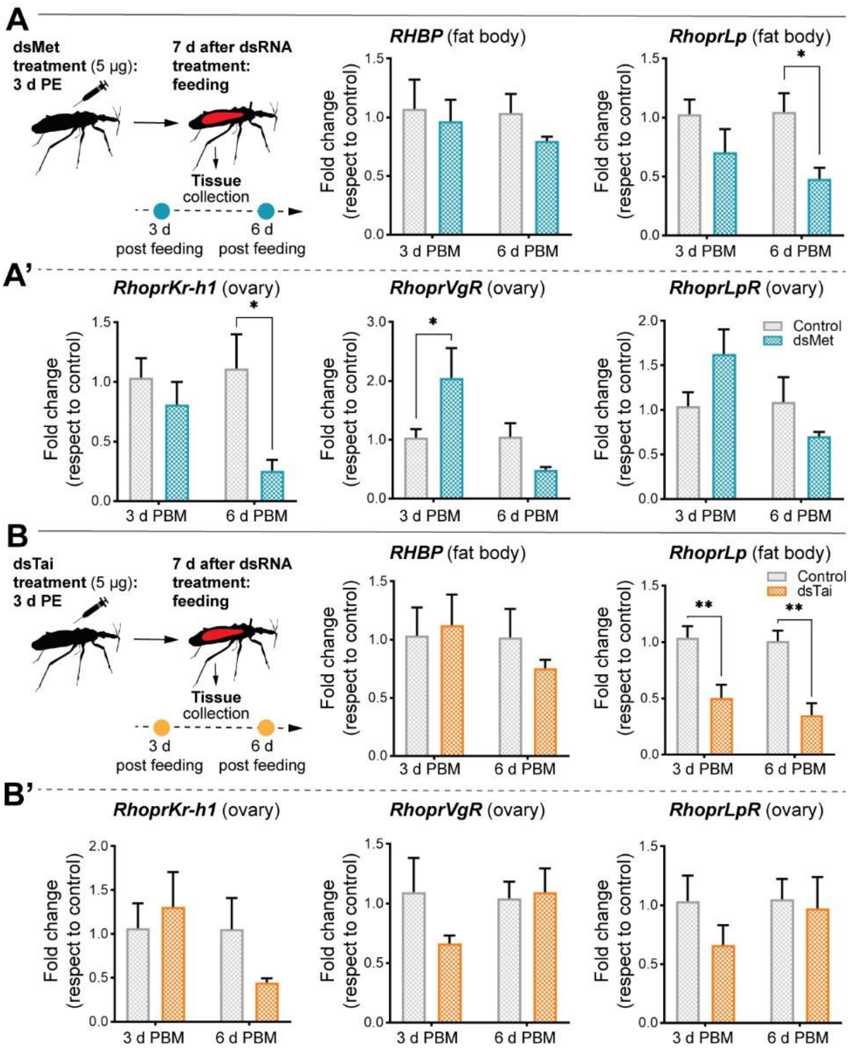

Supplementary Figure S5. Effect of dsRNA treatment at two time points during vitellogenesis. (A) *RHBP* and *RhoprLp* mRNA expression in the fat body after dsMet injection. **(A')** *RhoprKr-h1, RhoprVgR* and **RhoprLpR** mRNA expression in the ovaries after dsMet injection. **(B)** *RHBP* and *RhoprLp* mRNA expression in the fat body after dsTai injection. **(B')** *RhoprKr-h1*, *RhoprVgR* and *RhoprLpR* mRNA expression in the ovaries after dsTai injection. Transcript levels were quantified using RT-qPCR and analyzed by the $2^{-\Delta\Delta Ct}$ method. The y axes represent the fold change in expression relative to control (dsARG, value ~ 1) obtained via geometric averaging using *Rp49* and *actin* as reference genes. The results are shown as the mean ± SEM (n = 5-6, where each n represents an individual tissue from 1 insect). *p<0.05 (Student's t-test).

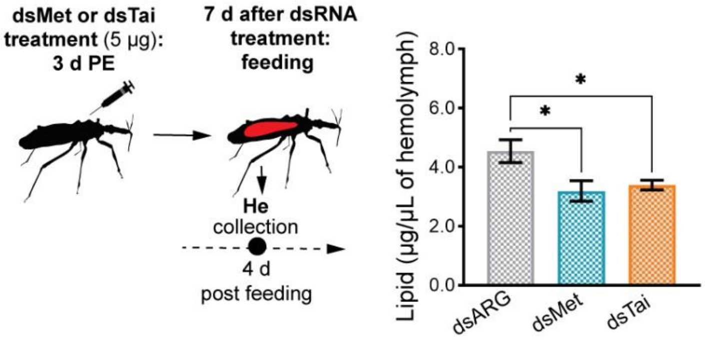

Supplementary Figure S6. Effect of dsRNA treatment on hemolymph (He) lipid levels in adult females at 4 days post blood meal. The y axis represents µg of lipid per µL of hemolymph. The results are shown as the mean ± SEM (n = 5-6, where each n represents hemolymph from 1 insect). *p<0.05 (One-way ANOVA and Tukey's test as the post hoc test).

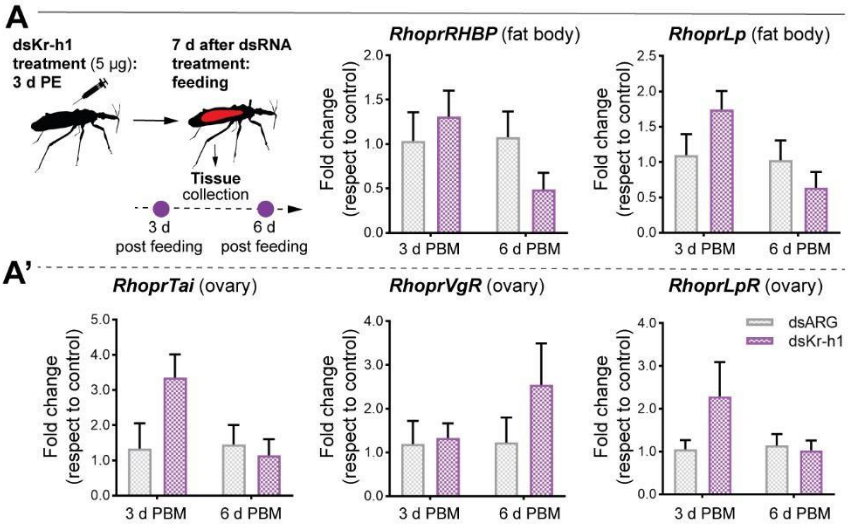

Supplementary Figure S7. Effect of dsKr-h1 treatment at two time points during vitellogenesis. **(A)** *RHBP* and *RhoprLp* mRNA expression in the fat body after dsKr-h1 injection. **(A')** *RhoprTai*, *RhoprVgR* and *RhoprLpR* expression in the ovaries after dsKr-h1 injection. Transcript levels were quantified using RT-qPCR and analyzed by the $2^{-\Delta\Delta Ct}$ method. The y axes represent the fold change in expression relative to control (dsARG, value ~ 1) obtained via geometric averaging using *Rp49* and *actin* as reference genes. The results are shown as the mean ± SEM (n = 5-6, where each n represents an individual tissue from 1 insect). *p<0.05 (Student's t-test).

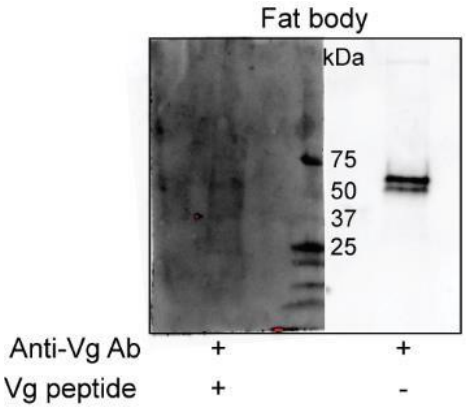

Supplementary Figure S8. Pre-adsorption ("blocking") control. The anti-vitellogenin antibody (Anti-Vg Ab, 1:2000 dilution in TBS-0.1% Tween 20 containing 5% BSA) was pre-incubated overnight at 4°C with a molar excess of the immunogen (Vg peptide: PLPQFVLQSRPELVPLPKLVAGGQVLDIVKTKNYSNCEQRMAYHFGLTGLTDWEPASNQ, final concentration: 1ng/μl). Pre-adsorption (left panel) substantially decreased the intensity of Vg staining in fat body protein; note an increase in the dark background due to overexposure for the low signal.

Supplementary Table S1. Primers used for qPCR and dsRNA synthesis.

Gene code	Primers to qPCR	Sequence (5❼3)
JN416985.1	[†]Met isoform 1_forward	CTAAGTCTACAAAGGAAGTCAC
	Met isoform 1_reverse	CCTGACGAAGTGACAATC
JN416985.1	Total Met_forward	GATGGGAGGAATCCTGTTGA
	Total Met_reverse	GGCAAGTCTGGATCGAAGTC
RPRC001318; RPRC001317	Tai_forward	CACAACGTCCATCCACTCCA
	Tai_reverse	TTCTTTGCAGCGGTCTCACT
RPRC014398	Kr-h1_forward	ACAACCTGTAGTGGCTGTCG
	Kr-h1_reverse	CGTACACTGTAGCGTGTCGT
RPRC013511	Vg 1_forward	TTGCTAGTCGCATGAACCTG
	Vg 1 _reverse	TTTAGTGGTGCATCGCTCTG
RPRC002109	Vg 2_forward	TCCATTGCCTAACCTCCTTG
	Vg 2_reverse	GTAAGGACGATGCGGCTAAC
RPRC002125	Lp_forward	CTTTGCACATCGGAGACTGA
	Lp _reverse	GTTCCCTGTATGCGCATTTT
RPRC009875	Actin_forward	AGAGAAAAGATGACGCAGA-TAATGT
	Actin _reverse	ATATCCCTAACAATTTCAC-GTTCG
RPRC014419	Rp49_forward	GTGAAACTCAGGAGAAATTGGC
	Rp49_reverse	AGGACACACCATGCGCTATC
AJ421962.1	18S_forward	TCGGCCAACAAAAGTACACA
	18S_reverse	TGTCGGTGTAACTGGCATGT
RPRC011390	LpR_forward	CTCGATGAACCGAGAGCAAT
	LpR_reverse	ATTCAGTTTGGCGTCTACCC
RPRC000551	VgR_forward	ATTTGGACGGATTGGGGTA
	VgR_reverse	TGGAGGAAAGAATGGTCCTG
RPRC004408	RHBP_forward	TCCTTCACACTCTCCGCAAC
	RHBP_reverse	GTACGCTTGGTACGCCACTT
RPRC007496	Thiol_forward	CAAAGTTAATGTACAC-GGTGGTG
	Thiol_reverse	CTCCAGACTTCAACGCTGTTA

RPRC007884	§HMGS_forward	GCAACTGTTTGAA-GAAAGTGGTA
	HMGS_reverse	AAGCACTGGTACCTCCAAAG
Supercontig RproC3:KQ03422 6 minus strand 878233-875494	§HMGR_forward	GGCATAGAAAGAAGATGAC-CAAAC
	HMGR_reverse	GCACGAGTATCAAGACAACAA-TATG
RPRC014277	§MEVK_forward	GAAAGATCAAGAGGAACGAG-GAG
	MEVK_reverse	CGCTTATGTGAGACAC-CTAATGAT
RPRC010547	§FOLD_forward	AAACCGAGCGATGTTGT
	FOLD_reverse	GTAGGTTGGATAACTAG-TTCTGAT
RPRC002910	§FALDH_forward	AGTACCTTACAGTCTAGTATTT-GCC
	FALDH_reverse	GATCTGTCTTCAGCACCGTT
RPRC011659	§JHAMT_forward	GGACCAGGCGATGTTACTTT
	JHAMT_reverse	CCAAATCATCAGAAA-TATCGCTTCC
RPRC000513	§Epox_forward	CGGAGAATTGAT-TCATGATGATTGG
	Epox_reverse	GTAACGGCGGTGACAGTAAA
**Gene code**	**Primers to dsRNA synthesis**	**Sequence (5❼3)**
RPRC014398	dsKr-h1a_forward	**TAATACGACTCACTATAGGGA-GA**ACA ACAAGTGGTAGCGGTGT
	dsKr-h1a_reverse	**TAATACGACTCACTATAGGGA-GA**TTCT CACCTGTATGCGTCCG
RPRC014398	dsKr-h1b_forward	**TAATACGACTCACTATAGGGA-GA**CTTG TGCGTCTTCAACCAGC

	dsKr-h1b_reverse	**TAATACGACTCACTATAGGGA-GA**CTA CTCGGCGGGGTTAACAG
RPRC001318	dsTai a_forward	**TAATACGACTCACTATAGGGA-GA**CGC TTTTCCTTTCAGCCCAC
	dsTai a_reverse	**TAATACGACTCAC-TATAGGGAGA**CAG CTTCTTCGGAACTCGGT
RPRC001318	dsTai b_forward	**TAATACGACTCACTATAGGGA-GA**GCT CCGCCAAGAAATCGAAC
	dsTai b_reverse	**TAATACGACTCACTATAGGGA-GA**TAA GCTGCCGAAATGCCAGA
JN416985.1	dsMet a_forward	**TAATACGACTCACTATAGGGA-GA**TAA TTGGTGCCATTGCGTGC
	dsMet a_reverse	**TAATACGACTCACTATAGGGA-GA**AAT CATCACCCACCGCACAT
JN416985.1	dsMet b_forward	**TAATACGACTCACTATAGGGA-GA**AGC GCAGATGAAAGCTCAGT
	dsMet b_reverse	**TAATACGACTCACTATAGGGA-GA**CCC ATAACCGCGGGTCAATA
ARG	dsARG_forward	**TAATACGACTCACTATAGGGA-GA**ATG AGTATTCAACATTTCCGTGTC
	dsARG_reverse	**TAATACGACTCACTATAGGGA-GA**AAT AGTTTGCGCAACGTTG

***TAATACGACTCACTATAGGGAGA =** T7 RNA polymerase promotor

†Primers reported by Villalobos-Sambucaro et al. [21]

§Primers reported by Villalobos-Sambucaro et al. [6]

Abbreviations: Met, Methoprene-tolerant; Tai, Taiman; Kr-h1, Krüppel-homolog 1; Vg, vitellogenin; Lp, lipophorin; Actin, β-actin; Rp49, 60S ribosomal protein L32; 18S, 18S ribosomal RNA; LpR, lipophorin receptor; VgR, vitellogenin receptor; RHBP, Rhodnius heme binding protein; thiol, Acetyl-CoA-thiolase; HMGR, HMG-CoA reductase; HMGS, HMG-CoA synthase; MEVK, Mevalonate Kinase; FOLD, Farnesol dehydrogenase; FALDH, Farnesol dehydrogenase; JHAMT, Juvenile hormone acid methyltransferase; Epox, Methyl farneseoate epoxidase; ARG, ampicillin resistance gene; ds, double stranded

1. Villalobos-Sambucaro, M.J.; Nouzova, M.; Ramirez, C.E.; Alzugaray, M.E.; Fernandez-Lima, F.; Ronderos, J.R.; Noriega, F.G. The juvenile hormone described in *Rhodnius prolixus* by Wigglesworth is juvenile hormone III skipped bisepoxide. *Sci. Rep.* **2020**, *10*, 3091.
2. Villalobos-Sambucaro, M.J.; Riccillo, F.L.; Calderón-Fernández, G.M.; Sterkel, M.; Diambra, L.A.; Ronderos, J.R. Genomic and functional characterization of a methoprene-tolerant gene in the kissing-bug *Rhodnius prolixus*. *Gen. Comp. Endocrinol.* **2015**, *216*, 1–8.
